# Stiffness Dependent Separation of Cells in a Microfluidic Device

**DOI:** 10.1371/journal.pone.0075901

**Published:** 2013-10-16

**Authors:** Gonghao Wang, Wenbin Mao, Rebecca Byler, Krishna Patel, Caitlin Henegar, Alexander Alexeev, Todd Sulchek

**Affiliations:** 1 Woodruff School of Mechanical Engineering, Georgia Institute of Technology, Atlanta, Georgia, United States of America; 2 Wallace H. Coulter Department of Biomedical Engineering, Georgia Institute of Technology, Atlanta, Georgia, United States of America; 3 Petit Institute for Bioengineering and Bioscience, Georgia Institute of Technology, Atlanta, Georgia, United States of America; University of Cambridge, United Kingdom

## Abstract

Abnormal cell mechanical stiffness can point to the development of various diseases including cancers and infections. We report a new microfluidic technique for continuous cell separation utilizing variation in cell stiffness. We use a microfluidic channel decorated by periodic diagonal ridges that compress the flowing cells in rapid succession. The compression in combination with secondary flows in the ridged microfluidic channel translates each cell perpendicular to the channel axis in proportion to its stiffness. We demonstrate the physical principle of the cell sorting mechanism and show that our microfluidic approach can be effectively used to separate a variety of cell types which are similar in size but of different stiffnesses, spanning a range from 210 Pa to 23 kPa. Atomic force microscopy is used to directly measure the stiffness of the separated cells and we found that the trajectories in the microchannel correlated to stiffness. We have demonstrated that the current processing throughput is 250 cells per second. This microfluidic separation technique opens new ways for conducting rapid and low-cost cell analysis and disease diagnostics through biophysical markers.

## Introduction

Rapidly sorting and separating cells are critical for detecting diseases such as cancers and infections and can enable a great number of applications in biosciences and biotechnology. For example, diseased cells have been identified through morphological differences with healthy cells, and fluorescent molecular markers are routinely used to separate specific subpopulations of cells [1], [2]. However, the morphological overlap between the diseased and healthy cells often poses a significant problem to accurate identification of cell populations. New molecular and biophysical markers which can be readily detected and used to rapidly sort cells are vital for improving separation of different cell subpopulations and accurately detecting specific disease conditions.

A variety of different physical mechanisms have been used to separate cells, including magnetic fields [3]–[5], electric fields [6]–[9], optical forces [10]–[12] and acoustic fields [13]–[15]. However, these active separation methods require an external field which adds to the complexity and increases the cost. Alternatively, labeling of cells through specific binding of fluorescent antibodies [16] is expensive, requires highly-trained personnel, and hampers the downstream analysis of separated cells. Additionally, the separation executed by these techniques occurs only after individual readout of the labeling differentiation which limits the throughput.

Consequently, a label-free method that can separate cells continuously by biophysical properties would greatly complement existing separation technologies. While a variety of techniques demonstrate separation by physical parameters such as size [17], mass [18], and adhesion [19], a straightforward method to separate cells by mechanical stiffness would benefit biomedical capabilities. A number of pathophysiological states of individual cells result in drastic changes in stiffness in comparison with healthy counterparts. Mechanical stiffness has been utilized to identify abnormal cell populations in detecting cancer [20]–[22] and identifying infectious disease [23]. For example, several studies have shown a reduction in cell stiffness with increasing metastatic efficiency in human cancer cell lines [23]–[25]. Recently, microfluidic methods were developed to classify and enrich cell populations utilizing mechanical stiffness [26]–[31]. One problem with these methods is an overlap between the natural variations of different biophysical properties that can influence stiffness-based separation, such as variations in size [28], [32], [33] and optical refractive index [24].

**Figure 1:**
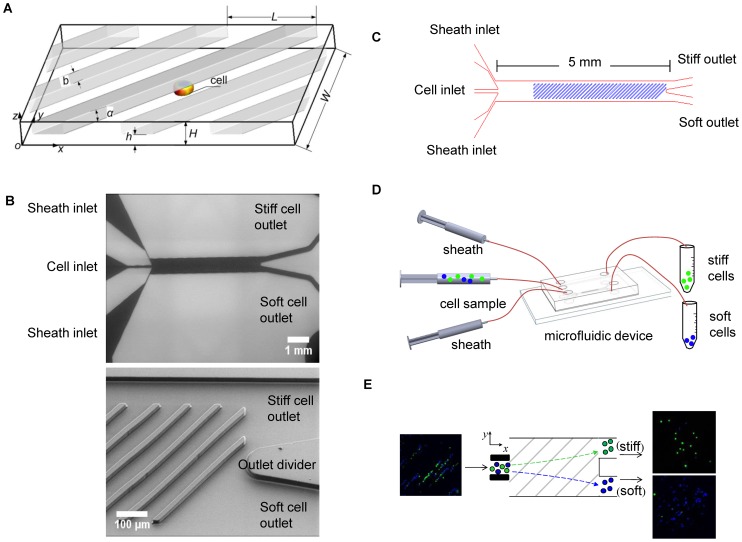
Schematics of the microfluidic device and the experimental setup for the cell separation process. (A) Schematic of channel dimensions. The channel height is 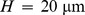. The ridge is inclined at angle 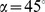 and the ridge width 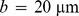. The spacing 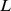 of ridge period is 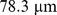. The gap 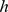 between the ridges and the substrate is varied depending on cell stiffness. (B) SEM images of the permanent mold (top) and microfluidic device made of PDMS showing ridges and outlet divider (bottom). (C) Schematic of a microfluidic channel. The channel is 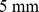 in length and 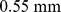 in width. (D) Schematic of the experimental setup for cell separation process. A mixture of two types of cells with difference in stiffness is input into the device and the separated cells are collected at two outlets. (E) Images of separated cells with nuclear cell stains illustrate untreated K562 cells (green) and 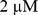 cytochalasin D softened K562 cells (blue) undergoing separation.

In this paper, we demonstrate a new strategy to continuously and non-destructively separate cells into subpopulations by exploiting the variation in mechanical stiffness between individual cells. In our microfluidic separation method, we employ a microchannel with the top wall decorated by a periodic array of rigid diagonal ridges (Figure 1A). The microchannel with ridges are micro-fabricated (Figure 1B) and designed to include sheath flows to focus the cells in the center of the channel and two outlets for stiff and soft cells (Figure 1C). The gap between the ridges and the bottom channel wall is smaller than the cell diameter, thus the cells streamed through the channel are periodically compressed by the ridges to effectively “probe” the cell mechanical stiffness. The difference in mechanical resistance to compression of cells with different stiffness gives rise to a stiffness-dependent force associated with cell passage through constrictions formed by the consecutive channel ridges. This elastic force is directed normal to the compressive diagonal ridges and, therefore, has a component that deflects cells propelled by the flow in the transverse direction with a rate proportional to their stiffness. In addition to the elastic force, cells experience a transverse hydrodynamic force due to circulatory flow created by diagonal ridges. The elastic and hydrodynamic forces act in the opposing transverse directions and the balance between these two forces sets cell trajectories that rapidly diverge for cells with different stiffness. We employ this principle to separate cells with dissimilar mechanical stiffness, as shown schematically in Figure 1D. We demonstrate that this method can be operated at 250 cells per second while processing a cell concentration of one million cells per mL (Figure 1E), where cells that were chemically softened (stained blue) were separated from untreated cells (stained green) after mixing. We find that the separation results are weakly sensitive to natural variations in cell size found within cell lines.

The paper is organized as follows. We first seek to show that cells of different stiffnesses, but are otherwise similar in physical properties, follow different trajectories within the device and can therefore be sorted. Secondly, we examine the physical mechanism of separation through computer simulations which are directly compared to our experimental separation results. Then, we show that different cell types, which are of similar sizes but different stiffnesses, can be mixed and preferentially enriched at the output of the microfluidic device with diagonal ridges. The separation results include cells that are artificially softened and stiffened through chemical treatments, as well as separation of epithelial cancer cells from similarly sized white blood cells. We also show that the separated cells indeed differ by stiffness by using atomic force microscopy measurements and discuss different parameters influencing cell sorting. Finally, we discuss the effect of different channel parameters on separation results and present our conclusions.

## Results and Discussion

### Variation in Cell Trajectory Due to Stiffness

**Figure 2:**
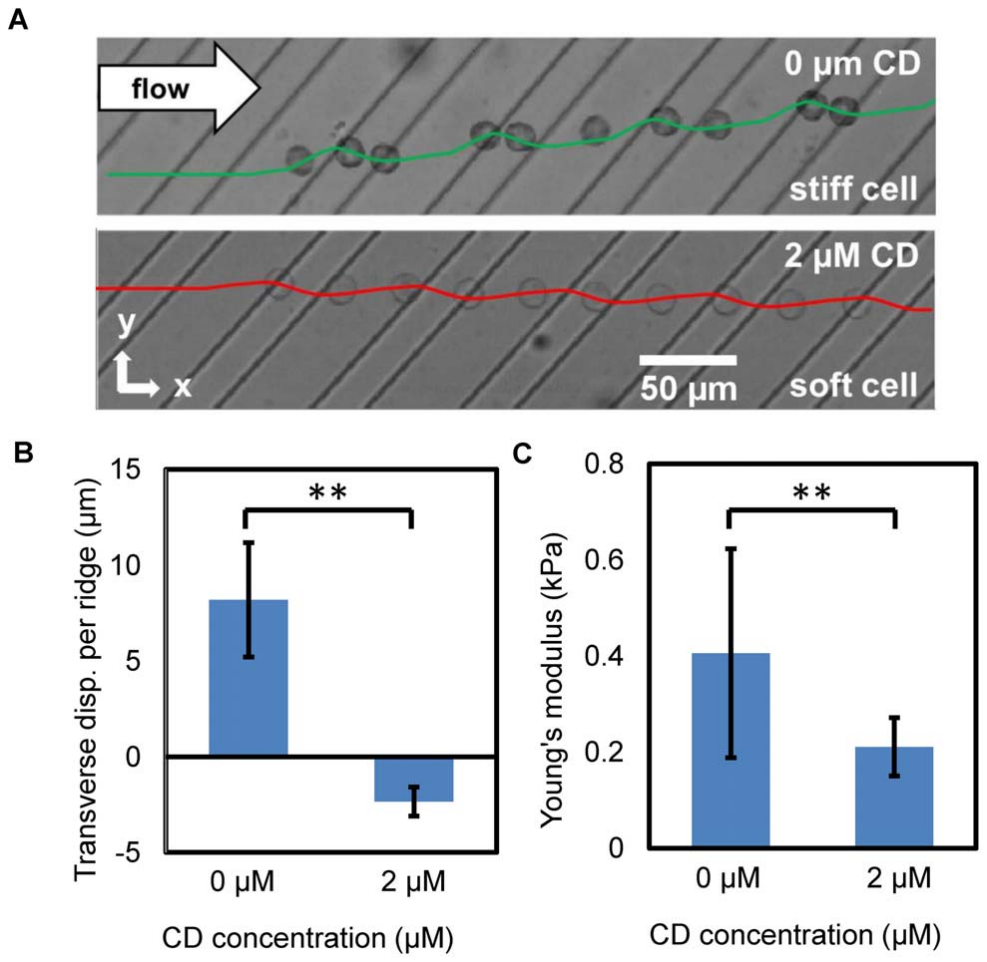
Cell trajectories are a function of cell stiffness. (A) Overlay of still frames from a video of an untreated and 2 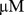 CD softened K562 cells flowing in a channel. Each micrograph is an overlay of 10 still frames at equal 10 ms time intervals from a video taken at 1200 fps. Green and red solid lines represent numerical simulations of the flow trajectory stiff and soft capsules. (B) Cell transverse displacement per ridge (n = 110 cells for each cell population) for untreated K562 cells and 2 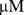 CD softened K562 cells are 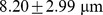 and 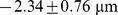 respectively. (C) Young's modulus (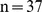 for each cell population) for untreated K562 cells and 2 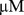 CD treated K562 cells are 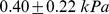 and 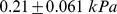 respectively. The error bars represent the standard deviation. Nonparametric Wilcoxon signed-rank tests were used to test statistical significance between the two cell populations, with ** indicating a p<0.0001.

To demonstrate the stiffness-based cell separation, we fabricated a ridged microfluidic channel using standard replica molding (For details see “Materials and Methods” and Figure S1). We first examined the cell trajectories of K562 lymphoblastic cells in the ridged microfluidic channel (Figure 2A). K562 cells were chemically softened using actin depolymerizing agent cytochalasin D (CD) to create cell subpopulations that only differ by their mechanical stiffness. Untreated (stiff) K562 cells and 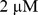 CD softened (soft) K562 cells were separately flowed through the microfluidic channel.

Figure 2A shows a collection of high-speed video still frames of a single untreated K562 cell and a 2 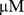 CD softened K562 cell flowing through the microfluidic channel. The micrographs of the cells are overlayed at equal time intervals (10 ms). The video microscopy revealed that the stiffer cell has a tendency to move in the direction parallel to the ridge, resulting in a net positive transverse displacement perpendicular to flow direction (see also Movie S1). After softening of K562 cells with 2 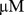 CD, the transverse displacement became negative (Movie S2). The transverse displacement per ridge for untreated K562 cells and 2 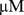 CD softened K562 cells was found to be 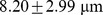 and 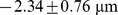 respectively (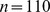 cells for each cell population) and is displayed in Figure 2B. The error bars represent the standard deviation. A nonparametric Wilcoxon signed-rank test was used to show that the separated populations were significantly enriched for both cell types.

To verify that the CD treated K562 cells were indeed mechanically softened, AFM measurements were conducted on identical cell populations and showed a decrease in Young's modulus from 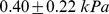 to 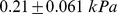, (Figure 2C, with
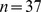
cells for each cell population). The large standard deviation is due to the natural cell stiffness variation within the cell population. The addition of CD has effectively decreased the Young's modulus of K562 cells by 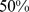, which is in agreement with previous studies [34], [35]. The ability to separate cells with a decrease of 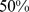 in Young's modulus is relevant to biomedical investigations as this stiffness decrease is similar to softening seen in metastatic cancer cells [20].

### Cell Separation Principle

To investigate the physical principle that results in cells of different stiffnesses to separate within the microfluidic channel, we performed numerical simulations of deformable fluid-filled capsules flowing in a ridged microchannel. Figure 2A shows the simulated spatial trajectories of centers of mass of stiff and soft capsules overlayed with the experimentally observed trajectories of a stiff and soft cell respectively. The simulation shows that compliant capsules having difference in stiffness exhibit diverging trajectories that are in agreement with cell trajectories observed in the experiments. We will employ our computational model to examine the separation principle in detail.

**Figure 3:**
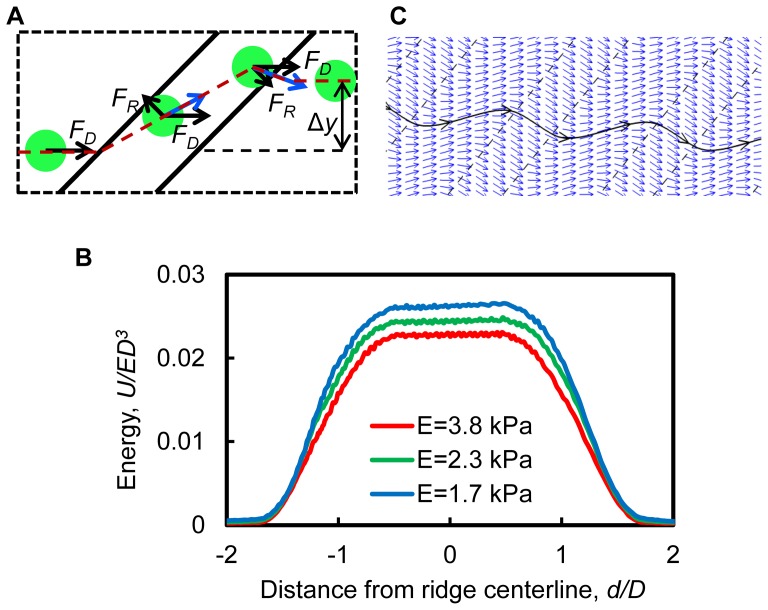
Numerical simulations that demonstrate the separation principle. (A) Cells experience both a hydrodynamic force, 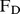, and an elastic force, 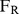, as the cells are deformed by the ridges. The elastic force varies with cell stiffness. The net transverse displacement is a result of interplay between the hydrodynamic force and stiffness-dependent elastic force. (B) The free energy associated with cell compression, 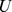, increases to a maximum as the cell passes through the ridge and varies as a function of cell Young's modulus. The difference in the gradient of free energy of soft and stiff cells gives rise to different transverse forces that deflect cell trajectories in the microchannel perpendicular to the ridge and dependent on cell mechanical stiffness. (C) Simulation of velocity field and the resulting streamlines. The diagonal ridges create secondary flows (blue arrows represent velocity vector of the flow) that circulate underneath the ridges which propels soft cells in the negative transverse direction. The trajectory of soft cells follows closely to the streamline due to the minimal elastic force.

Cells propelled by fluid flow experience a hydrodynamic (drag) force due to the viscous fluid 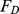 and an elastic force 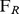 when they confront periodic ridges in a microchannel (Figure 3A). This transversal force arises due to cell deformation and, therefore, is proportional to the cell stiffness. Thus, cells with different stiffness experience different elastic forces as they pass through periodical constrictions. Thermodynamically, this elastic force is associated with the gradient of system free energy due to cell elastic deformation (see Figure 3B for the free energy of capsule compression for 3 representative stiffnesses) and, therefore, is in the direction perpendicular to the ridge. Since the ridges are oriented with an angle relative to the bulk fluid flow, this force is not aligned with the flow direction, but rather has a component that displaces cells normal to the flow (Figure 3A). The elastic force has opposite directions when a cell enters and leaves a constriction. However, diagonal ridges create an asymmetry in cell trajectory that results in a net transversal displacement. Stiffer cells experience a larger elastic force and, thus, greater transversal displacement in the positive transverse direction.

When cells are soft, the elastic force is weak and cells move with fluid flow streamlines. Diagonal ridges create a flow circulation in the microchannel, in which the fluid near the channel bottom flows in the negative transverse direction (Figure 3C) [36], [37]. Since cells in the stream are located near the bottom channel wall (Figure 1A), soft cells are transported by the circulating flow in the negative transverse direction. As a result, soft and stiff cells migrate to opposite sides of the ridged microchannel, thereby separating according to their mechanical stiffness.

### Cell Separation in the Microfluidic Device

The primary goal of this study is to demonstrate separation of cell populations which are of similar size but of different stiffness. We chose to investigate cell lines which simulate the presence of epithelial cancer cells (HeyA8 and Hey) mixed with white blood cells (Jurkat and K562). The successful separations for these cell mixtures point to the potential for the device to be used for metastatic cancer screening and monitoring.

**Figure 4:**
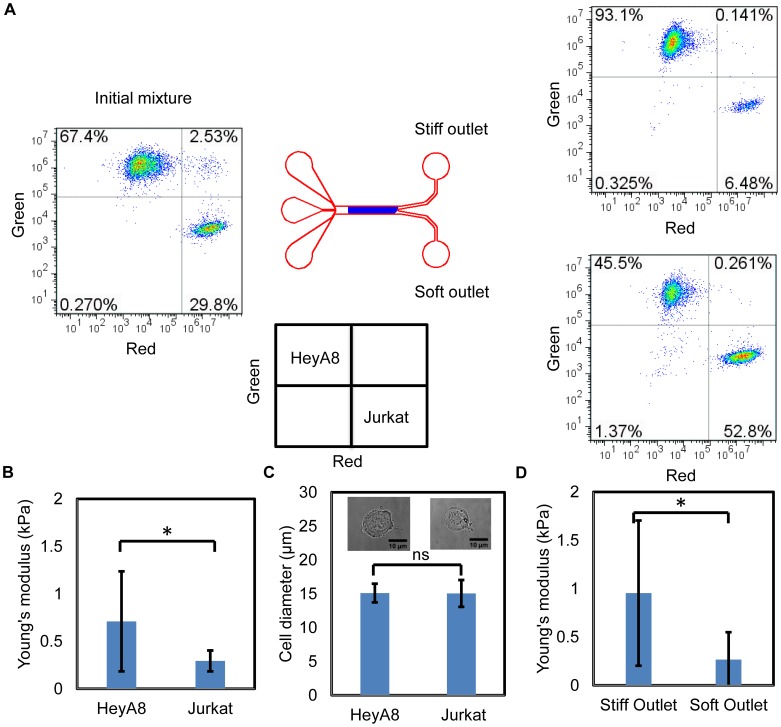
HeyA8 cells and Jurkat cells have similar cell diameters but different stiffnesses and can be separated. (A) Flow cytometry analyses of the initial mixture of cells and the cells collected at the stiff and soft outlets show the enrichment for HeyA8 cells (*E* = 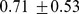 kPa) was 5.7-fold and for Jurkat cells (*E* = 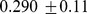 kPa) was 3.1-fold (*N* = 6). HeyA8 cells were fluorescently labeled green for these studies and Jurkat cells were labeled red. (B) AFM measurement of Young's modulus of Jurkat cells and HeyA8 cells initially, before mixing and flowing, show that HeyA8 cells and Jurkat cells differ greatly in Young's modulus (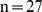
cells for each cell type). (C) HeyA8 cells and Jurkat cells are similar in cell diameter when suspended (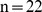, 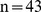 respectively). (D) Separated cells at outlets were measured by AFM (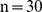 for each outlet). Nonparametric Wilcoxon signed-rank tests were used to test statistical significance, with * indicating a p<0.001 and ns indicating no significance.

Jurkat (*E* = 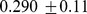 kPa, where *E* is the Young's modulus) and HeyA8 (*E* = 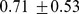 kPa) cells were labeled fluorescently, mixed and flowed through the microfluidic device. Separated cells were collected at the outlets. The separation result was verified with flow cytometry analysis (Figure 4A). Cell enrichment was observed at both the stiff and soft outlets. We observed 6.3-fold enrichment for HeyA8 cells and 3-fold enrichment for Jurkat cells (*N* = 6, where *N* is the number of independent experiments). We quantify cell separation in terms of enrichment which is defined by dividing the ratios of stiff and soft cells at outlets and inlets. For example, the enrichment for HeyA8 cells is 
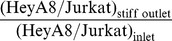
. We measured the Young's modulus of HeyA8 cells and Jurkat cells before the flow experiment and found they were significantly different (Figure 4B) while being of similar size (Figure 4C). Due to the large natural variation of stiffness within the cell population (Figure 4B), only partial collection of the stiff cells at the stiff outlet was observed. To confirm that the mixed cells were separated according to stiffness, we performed additional flow experiments with the mixed HeyA8 and Jurkat cells without any fluorescent labeling. Cells collected at the outlets were immediately measured with AFM and found to be of significantly different stiffnesses (Figure 4D). This result proves that similar sized cells from different cell lines can be separated into stiff and soft subpopulations.

**Figure 5:**
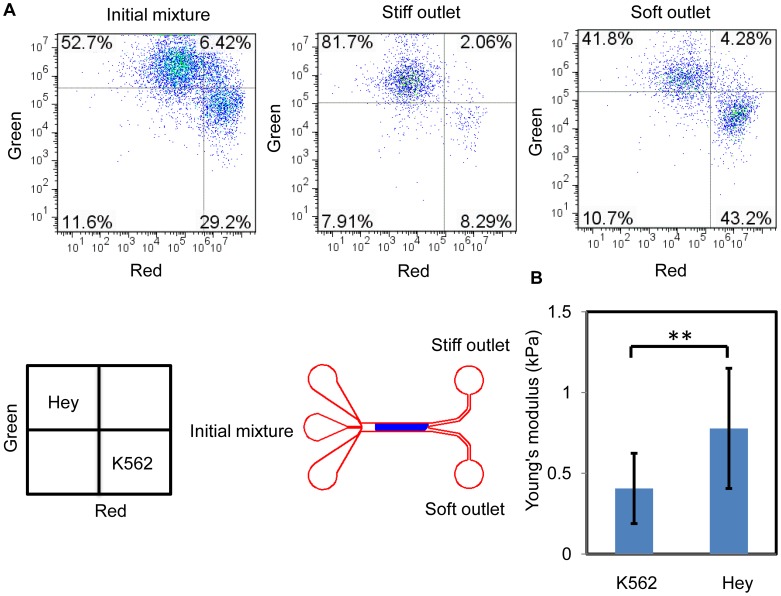
Hey cells and K562 cells separation. (A) Flow cytometry analyses of the initial mixture of cells and the cells collected at the stiff and soft outlets show an enrichment for Hey cells (*E = 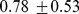
* kPa) of 5.3-fold and for K562 cells (*E* = 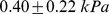) of 1.8-fold (*N* = 2). (B) Cell stiffness was measured with AFM (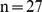
cells for each cell type) and quantified in terms of Young's modulus. A nonparametric Wilcoxon signed-rank test was used to test statistical significance, with ** indicating a p<0.0001.

To further demonstrate our cell separation method, we flowed a mixture of Hey cells (*E* = 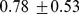 kPa) and K562 cells (*E* = 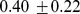 kPa) using the same flow conditions. The stiffness difference of this cell pair was less than 400 Pa with increased overlap between the two cell types. The separation results were evaluated by flow cytometry (Figure 5A) and enrichment for Hey cells was 5.3-fold at the stiff outlet and for K562 cells was 1.8-fold at the soft outlet (*N* = 2).

**Figure 6:**
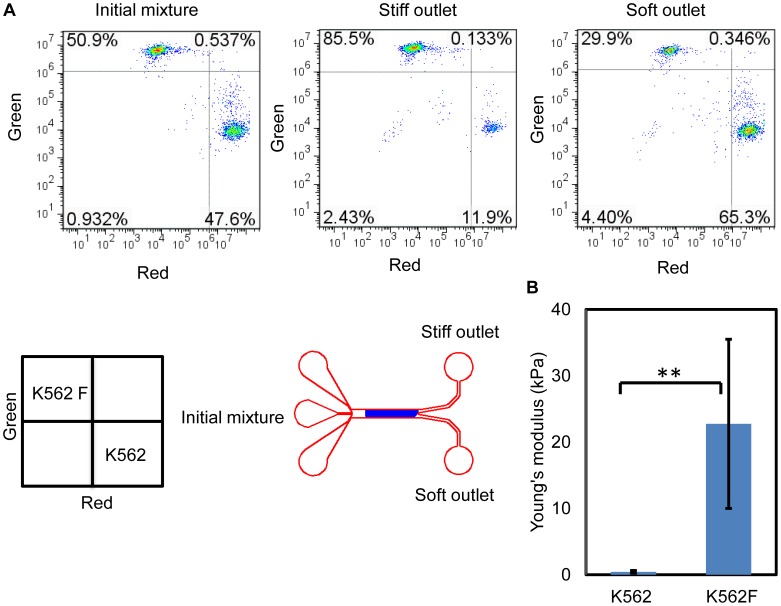
K562 cells and stiffened K562 cells separation. (A) Flow cytometry analyses of the initial mixture of cells and the cells collected at the stiff and soft outlets show an enrichment of both cell types at the stiff and soft outlet respectively. 4% formaldehyde treated K562 cells (*E* = 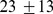 kPa) were enriched 6.7-fold at the stiff outlet and untreated K562 cells (*E* = 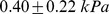) were enriched 2.3-fold at the soft outlet (*N* = 2). (B) Cell stiffness was measured with AFM and quantified in terms of Young's modulus. A nonparametric Wilcoxon signed-rank test was used to test statistical significance, with ** indicating a p<0.0001.

To examine the enrichment of cells with large differences in stiffness, we flowed a mixture of untreated K562 cells (*E* = 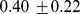 kPa) and 4% formaldehyde treated K562 cells (*E* = 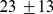 kPa) and analyzed the results with flow cytometry (Figure 6A, with treated cells denoted as K562F). The formaldehyde fixation process caused substantial cross-linking of cellular structures which produced cells with high stiffness, as measured by AFM (Figure 6B). The enrichment for formaldehyde treated K562 cells was 6.7-fold and for untreated K562 cells was 2.3-fold (*N* = 2). This result demonstrates that the ridged channel can be used to separate cells with large differences in stiffness.

To probe the lower resolution limitation of our microfluidic device, we flowed a mixture of Hey cells (*E* = 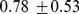 kPa) and HeyA8 cells (*E* = 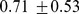 kPa) which have significant overlap in stiffness. Under the conditions tested, no appreciable separation between these two cell lines was achieved (data not shown). It is also possible to separate cells of different size and stiffness (for example, see Movie S3 showing the separation of epithelial cells from red blood cells), though size separation is outside the scope of this study.

To verify the viability of cells after flowing through the device, we cultured untreated K562 cells that were collected from the device outlets. The cells were cultured for six days and compared with a control population which did not undergo separation. We observed no significant difference in viable cell numbers and concluded that the majority of cells survived the transit and repeated compression through the device (Figure S2). Separated cells can therefore be used for downstream analysis.

### Cell Separation Parameters

Since cells within the same cell line vary in size, we examined the effect of size variation on stiffness sorting. An analysis of the correlation between cell stiffness, cell transverse displacement and cell diameter of individual cells revealed weak correlation for the natural variation that exists in the investigated cell lines (Table 1). In particular, K562 cell transverse displacement is weakly dependent on cell size as indicated by a low Pearson correlation coefficient. In addition, we found weak correlation between cell size and cell stiffness for Jurkat cells and HeyA8 cells (Table 1), a similar result has been reported previously [38].

**Table 1:** Cell size is weakly correlated to cell stiffness and cell transverse displacement.

Cell Types	Transverse Displacement Per Ridge Δy, (µm)	Cell Diameter d, (µm)	Pearson Correlation Coefficient Δy and d	Young's Modulus E, (kPa)	Cell Diameter d, (µm)	Pearson Correlation Coefficient E and d
**K562** **0 µM** **2 µM**	8.2±3.0–2.3±0.8	14.4±1.115.5±1.5	0.095–0.11	0.40±0.320.21±0.06	14.5±1.514.9±1.8	−0.150.34
**HeyA8**	–	–	–	0.71±0.53	16.8±2.6	−0.18
**Jurkat**	–	–	–	0.29±0.11	15.0±2.0	0.10

Pearson coefficients are used to measure the strength of correlation. Except for K562 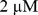 CD, which showed medium level of correlations, the other data showed weak correlations. The transverse displacement, cell diameter, and Young's modulus are represented as mean ± standard deviation. Sample size for K562 transverse displacement versus cell diameter is 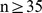 for each cell population. Sample size for K562 Young's modulus versus cell diameter is 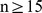 for each cell population. Sample size for HeyA8 and Jurkat Young's modulus versus cell diameter is 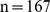 and 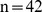 respectively.

The trajectory of cells in the microfluidic device is strongly affected by the size of the gap between the ridges and the bottom channel surface. The gap size 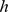 (Figure 1A) dictates the magnitude of deformation of the cell and, therefore, affects the cell trajectories, cell viability, and the probability of occlusion. In our cell flow experiments, we tested a range of gap sizes from 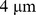 to 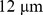. For the K562, HeyA8, Hey and Jurkat cells, we found that 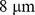 gap size was the best option for cell separation which minimized cell occlusion yet provided rapid separation for stiff and soft cells. The microfluidic channel with a gap greater than 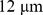 did not impose sufficient constriction, which led to small transverse displacement per ridge. On the other hand, a gap of less than 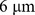 caused channel occlusion. In this case, the cells were seen to either roll along the ridges or become trapped under the ridges.

Flow rate is another key parameter in separation process since it defines the hydrodynamic force imposed on cells. The channel flow was formed by three inlet streams including two sheath streams which provided hydrodynamic focusing and a cell sample stream which contained the cells (Figure 1C). We investigated flow rate effect with Jurkat cells and HeyA8 cells. We observed that for high channel flow rate (greater than 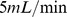) where hydrodynamic force was dominant, most of the cells migrated to the soft outlet and weak separation took place. On the other hand, a slow channel flow rate (less than 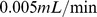) resulted in cells occluding the channel. The most efficient separation of Jurkat cells and HeyA8 cells occurred when the channel flow rate was about 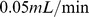 (Table 2).

**Table 2:** Effect of channel flow rate on Jurkat and HeyA8 cell separation.

	Enrichment			Cell Retention		
Channel Flow Rate	Stiff Outlet	Soft Outlet	Throughput	Stiff Outlet	Soft Outlet	Total
**0.025 mL/min (** ***N*** ** = 3)**	3.4-fold	2.1-fold	83 cells/sec	26%	26%	52%
**0.05 mL/min (** ***N*** ** = 6)**	6.3-fold	3-fold	250 cells/sec	41%	41%	82%
**0.25 mL/min (** ***N*** ** = 2)**	2.8-fold	1.1-fold	833 cells/sec	12%	72%	84%

*Numbers presented represent the averages of the trails conducted.*

At fast flow rate, the hydrodynamic force was dominant and pushed stiff cells migrated to soft outlet which resulted in the lowest enrichment. At slow flow rate, cells were stuck to the ridges and occluded the channel which resulted in the lowest throughput. We determined channel flow rate at 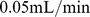 provided the best separation result. The cell retention is defined by calculating the ratio of cells collected at the outlet and total number of cells injected in the inlet. For example, the stiff outlet retention is 
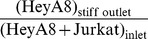
. The total retention is the sum of cells collected at the two outlets.

Other parameters that influence cell separation include ridge width, *b*, ridge angles, *α*, and ridge pitch, *L*. The ridge width should be comparable to cell diameter. If the ridge width is too small, the cells would only be deformed locally which results in a weak elastic force. On the other hand, if the ridge is too wide, the cell will have difficulty to pass through which leads to channel occlusion. Furthermore, the device should include a sufficient ridge pitch to allow the cells to mechanically relax. The larger the pitch, the more relaxation occurs and the transverse force/displacement should increase, though at a cost of increased channel length. The cell separation will also depend on the angle of ridges relative to the channel axis. The separation takes place due to simultaneous action of two opposing effects: compression of cells by ridges leading to the deflection of stiffer cells in the positive transverse direction, and circulatory secondary flows that transport softer cells in the negative transverse direction. The magnitude of the secondary flows is maximized when the ridges are oriented 45 degrees with respect to the channel axis. We therefore use this angle in our experiments and simulations to enhance the separation effect.

## Conclusions

We developed a new microfluidic approach to continuously separate cells by their differential stiffness. The operational principle of our separation method is based on the conversion of the difference in elastic energy of deformed cells with different stiffness into transverse displacement. The device has a simple design that includes a microfluidic channel with periodical diagonal ridges that are tilted with respect to channel axis and fabricated using standard photolithographic techniques. When cells are streamed through the device, they are periodic compressed by the diagonal ridges that then induce their transverse separation through differences in mechanical stiffness. The separated cells are then continuously collected at two device outlets. Cells do not require any special treatment or labeling. The cell separation process is passive and requires only pressure difference between inlets and outlets.

To demonstrate the operation of our device, we separated otherwise similar cells that differ in stiffness. We tracked the trajectories of untreated K562 cells and K562 cells softened with 2 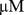 cytochalasin D. The trajectories were in agreement with our numerical simulation, substantiating our understanding of the physical principle. We also separated several cell lines with differences in stiffness and verified the enrichment with flow cytometry analysis and AFM measurements. We found that the natural variation in cell size examined in our study has weak effect on separation. Finally, we also demonstrated enrichment of cells having differences in both cell size and cell stiffness.

As such, a stiffness-based cell separation will be beneficial to process many cells, equivalent to conventional flow cytometry. The separation throughput depends on both the cell concentration and the flow rate. For cell lines used, we tested cell concentrations up to 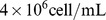 at which point the delayed transit times of stiffer cells at the leading edge of the ridge blocked the flow of subsequent cells, eventually leading to channel occlusion. Moreover, the flow rate will ultimately be limited by the decreased sensitivity to variations in stiffness due to the dominance of hydrodynamic force. For the results presented, a flow rate of 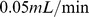 and cell concentration at 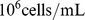 yielded a throughput of 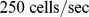. To further increase the throughput, a widened inlet stream can be used to process higher cell numbers with reduced cell-cell interactions.

To put this throughput in clinical context, a patient may have 10,000 white blood cells per 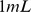 of blood. The microfluidic device can therefore process this amount of sample in less than one minute, potentially making this approach a powerful clinical tool for blood analysis. For example it may be employed to distinguish and enrich myeloid and lymphoid leukemia cells through biomechanical sorting [38], [39] without antibody labeling. Alternatively, differentiating and sorting invasive and noninvasive cancers [25] may improve cancer treatment. Furthermore, biomechanical enrichment of malaria infected red blood cells [40] may prove useful for lower detection limits of malaria for improved surveillance.

The continuous flow separation strategy has an advantage over previously reported methods [29], [30] in that sorted cells can be continuously collected at the outlets. Another major advantage is that our approach requires minimal external control and sample preparation. The stiffness-dependent separation is, therefore, important for the rapid enrichment of abnormal cells, which can enhance or potentially replace traditional disease detection methods. Thus, the use of the microfluidic platform for stiffness-based cell separation opens a way for rapid, low-cost biomedical assays and point-of-care clinical diagnostics.

## Materials and Methods

### Microfluidic Device Fabrication

The microfluidic device was made by replica molding Polydimethylsiloxane (PDMS) (Sylgard 184 Dow Corning Corp) on a permanent mold (Figure S1). The mold is made from SU-8 2007 through a two-step photolithography. The mold dimensions were measured with profilometry (Dektak 150 profiler) and verified with a confocal microscope (Olympus LEXT). The gap 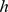 between the ridges and the bottom channel wall is designed to be smaller than the cell diameter (Figure 1A). Uncured PDMS was mixed in a 10∶1 ratio of elastomer to curing agent, then poured onto the SU-8 mold to a thickness of 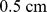 and cured in an oven at 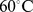 for 6 hours. The cured PDMS layer was peeled off the mold and inlet and outlet holes were punched with 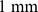 biopsy punch. The PDMS microchannel was treated with oxygen plasma (Harrick plasma cleaner PDS 32G) for 2 minutes then bonded to a glass slide. To prevent cell adhesion, bovine serum albumin (BSA) was dissolved in PBS at concentration of 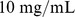 and the solution was injected into the channel and stored at 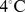 overnight.

### Cell Procurement and Preparation

The K562 cells (CCL-243) and Jurkat cells (CRL-1990) were purchased from ATCC. HeyA8 and Hey cell lines were provided by Dr. G. Mills (MD Anderson Cancer Center, Houston, TX) [41]. Red blood cells were withdrawn from healthy donors using protocols (H12002) approved by the Georgia Institute of Technology Institute Review Board. This research involving human participants was approved by the Georgia Institute of Technology Institute Review Board. We have received written consent from the donors of the RBCs used in this study. K562 cells were cultured and maintained in Iscove's modified Dulbecco's medium (ATCC) with the addition of 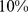 fetal bovine serum (FBS). Jurkat, Hey and HeyA8 cells were cultured and maintained in RPMI-1640 medium (Sigma) with the addition of 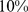 FBS. All cells were incubated at 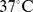 with 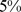 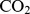. Cells were expanded to 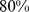 confluency in a culture flask over two days. CD was added to K562 cells and incubated for 2 hours and washed twice. To avoid the reversible effect of the CD treatment, the cells treated with CD were immediately used for the flow experiment and the AFM measurement. 4% formaldehyde was added to K562 cells and incubated at room temperature for 30 minutes and wished twice. RBCs were isolated from whole blood through centrifugation and 10% v/v sodium citrate anticoagulant was added and cell solution was diluted in PBS buffer. Four different cell labeling agents were used. Except for RBC experiment, we used lipid stains: Vybrant DiO (Life Technologies) and Vybrant DiD (Life Technologies) at 5 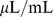. For the RBC experiment, we labeled HeyA8 cells with Vybrant Cell Tracer CFDA (Life Technologies) and RBCs with CellTracker CMTPX (Life Technologies).

### Experimental Setup

Syringe pumps (PHD 2000 Harvard Apparatus) were used to control the flow rates for the experiment. The flow experiment was carried out immediately after CD treatment. The schematic of the experimental setup is shown in Figure 1D. Cells inside the cell media were contained in a glass syringe and infused into the microfluidic device through polyethylene tubes. For CD treated K562 cell experiment, all cell trajectories were recorded within 2 hours after the CD treatment. The cell flow within the microfluidic device was observed with an inverted microscope (Nikon Eclipse Ti) and the high-speed videos were recorded using a high-speed camera (Phantom v7.3 Vision Research). In order to accurately capture the cell trajectories, we operated the high-speed camera at a minimum of 800 frames per second with a minimum resolution of 640 by 480 pixels for all videos and images. To prevent cell adhesion, 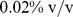 Tween 20 was added to sheath flow media. Separated cells were collected at the outlets and were analyzed with an Accuri C6 flow cytometer (BD Biosciences).

### Cell Stiffness Measurement with Atomic Force Microscopy

We utilized atomic force microscopy to accurately verify the stiffness of the cells. All cells were measured in suspended states with only slight attachment to the surface. To measure cells in suspended state, a monolayer of poly-l-lysine (MW 300,000 Sigma Aldrich) was grafted onto the glass slide substrate. This operation provided anchorage of the cell to the glass substrate while maintaining roundedness of morphology for cells and improved the cell stability during the AFM measurements. We carried out our AFM experiment immediately after the washing step and poly-l-lysine cell attachment treatment and all measurements were finished within 2 hours. We did not observe a change in measured stiffness during the course of these measurements. Measurements were conducted using a MFP-3D AFM (Asylum Research) attached to an inverted optical microscope (Nikon Eclipse Ti). A silicon nitride cantilever with a spring constant equal to 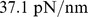 and a pyramidal tip was positioned above the center of a single cell and indented the cell. Prior work showed that the Young's modulus is a function of loading force and loading rate [42]. We utilized the same values for these parameters for all AFM measurements. The magnitude of indentation force used in all AFM measurements is 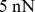 and the rate of indentation is 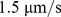. The applied force was sufficient to indent cells approximately 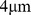. These values for the AFM parameters were selected to serve the purpose of comparing results with previous studies [38], [39], [42], [43]. The force-indentation curve was obtained for each measurement and then analyzed with a Hertzian model for a pyramidal tip (Wavemetrics, IgorPro software routines) from which the Young's modulus values were calculated.

### Cell Translation and Cell Size Measurement

High-speed videos were dissected into still frames using customized MATLAB codes. Cell diameters were measured using ImageJ. Cell translation was analyzed from stacks of still frames at equal-distance interval and was measured using ImageJ.

### Numerical Simulations

We used a hybrid method that integrates the lattice Boltzmann model [44], [45] (LBM) for the fluid dynamics and the lattice spring model [46], [47] (LSM) for the micromechanics of solids. The two models were coupled through appropriate boundary conditions at the solid-fluid interface [48], [49]. The details of our computational model and validation studies can be found elsewhere [36], [48], [50]–[53]. The dimensions of our periodic simulation box (Figure 1A) were: length 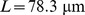, width 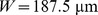, height 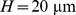 that corresponds to 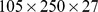 LBM nodes. The solid ridges were constructed using immobile LSM nodes arranged on a square lattice. The ridge geometry and gap size were identical to the experimental parameters. The deformable cell was modeled as a spherical fluid-filled elastic shell with a undeformed diameter 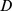. The shell was formed from one layer of 642 equally spaced LSM nodes connected by stretching and bending springs [54]. We set spring constants such that cell deformation matches cell elastic response during AFM experiment. A pressure gradient in the 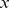 direction was imposed via a uniform body force to create Poiseuille flow in the channel. We applied non-penetration, no-slip conditions for the walls in the 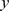 and 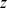 directions and a periodic boundary condition in the 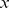 direction. Thus, we effectively modeled the motion of cells in a channel with a periodic array of diagonal ridges.

We characterized the flow in terms of the Reynolds number 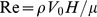 that represents the relative importance of inertial and viscous effects. Here, 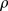 is fluid density, 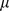 is fluid viscosity, and 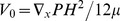 is the average fluid velocity in a straight channel of height 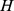 due to the pressure gradient 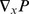. The magnitude of the Reynolds number is set to match the experimental value 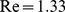. The cell interior is characterized by a high viscosity [55]. To mimic this property we set the viscosity of the fluid encapsulated in cell 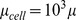. We started our simulations by placing a cell at the middle of the microchannel (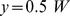) filled with a viscous fluid. We then imposed the pressure gradient on the initially motionless fluid and let the cell to move freely in the vertical plane, while keeping it from moving horizontally. When the flow was fully developed and the cell reached its equilibrium trajectory in the vertical plane, we released the cell so it could move freely with the flow in both the vertical and lateral directions. This procedure allowed us to eliminate the effect of the initial flow transient on the cell lateral migration and to analyze the dynamics of cells that start from identical position in the microchannel.

## Supporting Information

Figure S1
**Device fabrication sequence.** The device mold is made using the standard two-layer photolithography. Negative photoresist SU-8 2007 was spin-coated onto a 4 inch diameter silicon wafer. Uncured PDMS was poured onto the mold and allowed to cure in a 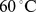 convection oven for 6 hours. The PDMS device layer was peeled off from the mold and inlet outlet holes were punched using a 1mm biopsy punch. Oxygen plasma was used to treat the PDMS device layer and glass slide for 2 minutes. Then, device layer was bonded to the glass substrate. The dimensions of the device were verified using a confocal microscope (LEXT Olympus).(TIF)

Figure S2
**K562 cell growth monitored for cells collected after flow experiment.** Cell concentrations were measured using a hemocytometer and recorded for seven days. The doubling time for entire seven-day observation was 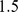 days for control and for 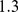 days for cells after flow experiment. Therefore, the growth rate (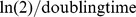) is 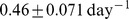 for the control (blue diamonds) and 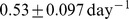 for the cells after flow experiment (red squares). The error bars represent standard deviations.(TIF)

Movie S1 Untreated K562 cells migrating through the microfluidic channel with positive y-displacement.
(AVI)

Movie S2

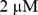
CD treated K562 cells migrating through the microfluidic channel with negative y-displacement.(AVI)

Movie S3 HeyA8 cells and red blood cells separation. The constriction gap is 10 µm. The video is taken at 800 frames per second.
(AVI)
