# Supplementary figures and images for: Stiffness Dependent Separation of Cells in a Microfluidic Device
Source: PLoS One. 2013 Oct 16;8(10):e75901. doi: 10.1371/journal.pone.0075901 (PMC3797716; doi:10.1371/journal.pone.0075901)

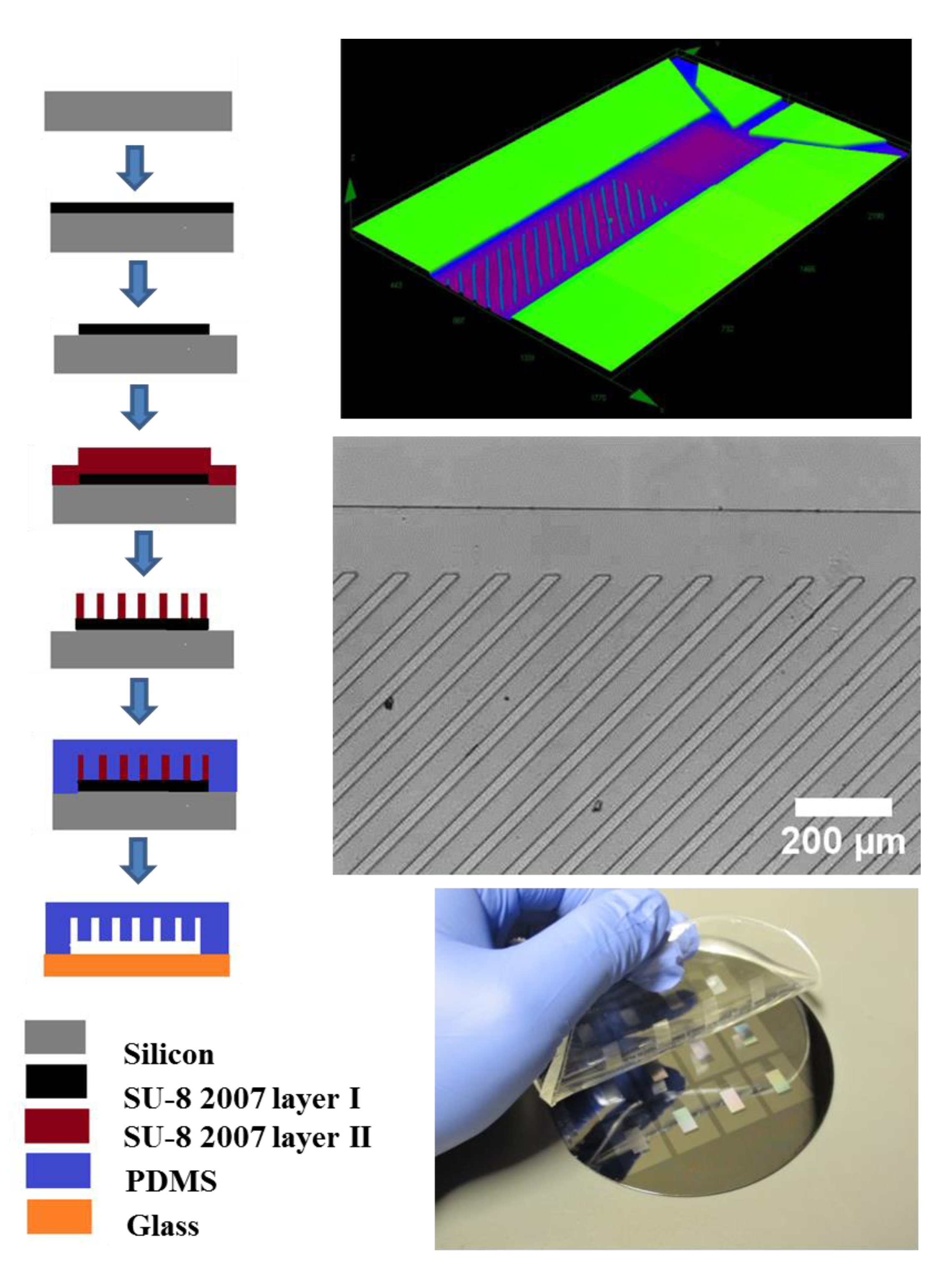

Supplement: Figure S1 — Device fabrication sequence. The device mold is made using the standard two-layer photolithography. Negative photoresist SU-8 2007 was spin-coated onto a 4 inch diameter silicon wafer. Uncured PDMS was poured onto the mold and allowed to cure in a convection oven for 6 hours. The PDMS device layer was peeled off from the mold and inlet outlet holes were punched using a 1mm biopsy punch. Oxygen plasma was used to treat the PDMS device layer and glass slide for 2 minutes. Then, device layer was bonded to the glass substrate. The dimensions of the device were verified using a confocal microscope (LEXT Olympus). (TIF) [file pone.0075901.s001.tif]

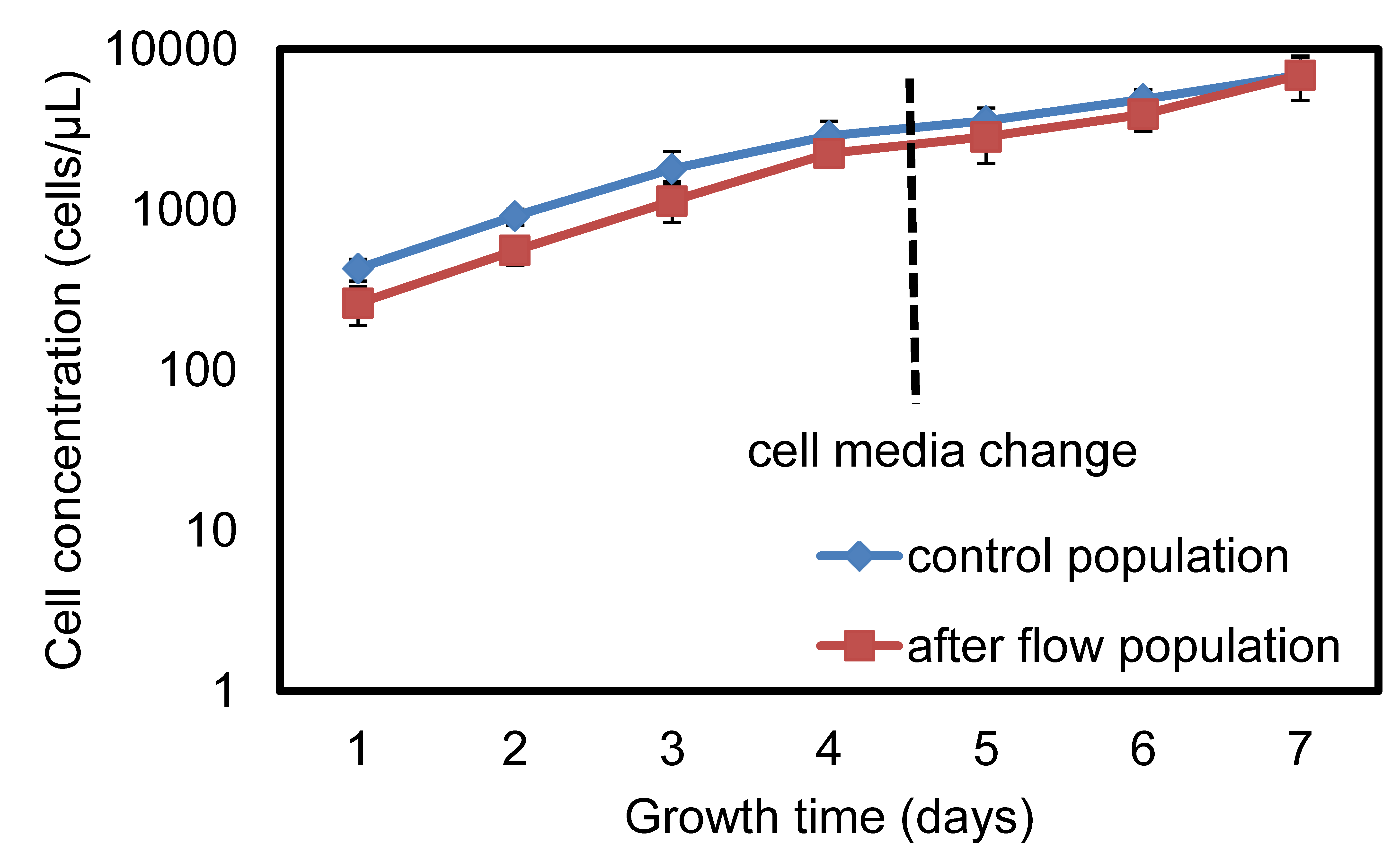

Supplement: Figure S2 — K562 cell growth monitored for cells collected after flow experiment. Cell concentrations were measured using a hemocytometer and recorded for seven days. The doubling time for entire seven-day observation was days for control and for days for cells after flow experiment. Therefore, the growth rate () is for the control (blue diamonds) and for the cells after flow experiment (red squares). The error bars represent standard deviations. (TIF) [file pone.0075901.s002.tif]
